# Effects of climate variation on bird escape distances modulate community responses to global change

**DOI:** 10.1038/s41598-021-92273-1

**Published:** 2021-06-18

**Authors:** M. Díaz, T. Grim, G. Markó, F. Morelli, J. D. Ibáñez-Alamo, J. Jokimäki, M.-L. Kaisanlahti-Jokimäki, K. Tätte, P. Tryjanowski, A. P. Møller

**Affiliations:** 1 Department of Biogeography and Global Change, Museo Nacional de Ciencias Naturales (BGC-MNCN-CSIC), c/Serrano 115bis, 28006 Madrid, Spain; 2 Department of Zoology and Laboratory of Ornithology, Palacky University, 77146 Olomouc, Czech Republic; 3 Behavioral Ecology Group, Department of Systematics, Zoology and Ecology, Eötvös Loránd University, Pázmány Péter sétány 1/c, 1117 Budapest, Hungary; 4 Department of Plant Pathology, Institute of Plant Protection, Hungarian University of Agriculture and Life Sciences, Ménesi út 44, 1118 Budapest, Hungary; 5 Faculty of Environmental Sciences, Community Ecology and Conservation, Czech University of Life Sciences Prague, Kamýcká 129, 165 00 Prague 6, Czech Republic; 6 Department of Zoology, Faculty of Sciences, University of Granada, 18071 Granada, Spain; 7 Nature Inventory and EIA-Services, Arctic Centre, University of Lapland, P. O. Box 122, 96101 Rovaniemi, Finland; 8 Department of Zoology, Institute of Ecology and Earth Sciences, University of Tartu, 19 51014 Tartu, Estonia; 9 Institute of Zoology, Poznań University of Life Sciences, Wojska Polskiego 71C, 60625 Poznań, Poland; 10 Ecologie Systématique et Evolution, Université Paris-Saclay, CNRS, AgroParisTech, 91405 Orsay, France

**Keywords:** Ecology, Zoology, Climate sciences, Environmental sciences

## Abstract

Climate and land use are rapidly changing environmental conditions. Behavioral responses to such global perturbations can be used to incorporate interspecific interactions into predictive models of population responses to global change. Flight initiation distance (FID) reflects antipredator behaviour defined as the distance at which an individual takes flight when approached by a human, under standardized conditions. This behavioural trait results from a balance between disturbance, predation risk, food availability and physiological needs, and it is related to geographical range and population trends in European birds. Using 32,145 records of flight initiation distances for 229 bird species during 2006–2019 in 24 European localities, we show that FIDs decreased with increasing temperature and precipitation, as expected if foraging success decreased under warm and humid conditions. Trends were further altered by latitude, urbanisation and body mass, as expected if climate effects on FIDs were mediated by food abundance and need, differing according to position in food webs, supporting foraging models. This provides evidence for a role of behavioural responses within food webs on how bird populations and communities are affected by global change.

## Introduction

Climate change, beside land-use change due to anthropization, is one of the main threats to global biodiversity and ecosystem functioning^1^. Efforts to understand climate effects usually focus on large-scale relationships among abiotic conditions and population trends, range shifts and phenological changes in the timing of migration and breeding^2–4^, following well-established niche-based approaches^5^. Climate-driven changes in biotic interactions have just recently been considered^6,7^, despite their key roles in the structure and dynamics of populations and communities. Recent analyses of relationships between climatic variables and community structure have shown clear-cut relationships between food web structure and climate change^8,9^. Direct trophic interactions govern many aspects of network dynamics, and here we propose that behavioural changes involved in such trophic interactions influence how species may respond to climate change.

Behaviour is the mechanism by which ecological effects are translated into resource acquisition, interactions between hetero- and conspecifics, and the transmission of resources into survival and reproduction. Surprisingly, behaviour has received little attention in studies of climate change^10^. Here, we propose using a robust metric of predator avoidance behaviour, that involves a trade-off between avoidance of danger while ensuring the acquisition of basic needs, to investigate how animals resolve this trade-off along spatial and temporal climate gradients, whose variation in temperature and precipitation reflects projected climate change. This approach follows the usual procedure of using current spatial variation as a proxy for future temporal changes^11^.

Predator avoidance behaviour can be estimated using the flight initiation distance (FID), i.e., the distance at which an animal flees when approached by a human under standardized conditions^12^. FID increases with fearfulness and varies predictably with environmental factors. FID is shorter in urban areas and those with more human activity^13–15^, lower predation risk^16^, lower food availability and higher physiological needs^17–19^. FIDs are repeatable within individuals and, presumably, heritable^20,21^, and they are closely correlated with species’ population trends and distributions^22–24^. FIDs are negatively related to population trends and range sizes under low levels of predation and disturbance (i.e. shorter FIDs enhance fitness in low-risk situations by allowing devotion of more time to foraging and reproductive activities), and positively under high-risk conditions, when predator avoidance is key for survival^22,23^.

Climate variation should affect FID in several ways. Warming reduces caloric needs, and increased precipitation elevates primary production and hence food availability, although warm temperatures may also decrease prey availability by decreasing hunting success^25,26^. Consequently, how birds adapt their FID to variation in temperature and precipitation should vary with the harshness and risk of their habitat conditions, their body size and their position in the trophic food web. These relationships allow us to make several predictions as to how FID should vary with climatic variation alone and in concert with other ecological factors. We expect (1) FID to be longer at higher temperatures, due to reduced caloric needs, and at increased precipitation, due to increased food availability, or FID to be shorter at high temperature and/or precipitation if reduced foraging success decreases food availability. Positive effects on food availability or reduced needs would increase FIDs, whereas interference in prey capture would decrease them. Furthermore, these effects of climate should be particularly strong under stressful conditions when the consequences of the acquisition of insufficient food are particularly dire. Thus, we expect (2) a positive interaction between climate variables and latitude such that increasing temperature or precipitation will have stronger effects on FID at higher latitudes, where abiotic conditions are more stressful^27^. FIDs are shorter in cities than in rural areas because cities have lower predation risk^13,16^, but also because cities may buffer effects of climate on food availability^17,18^. In addition, higher levels of human disturbance in cities have favoured adaptive reductions in FID^14^. Therefore, we expect (3) the effect of climate to be weaker within cities than in rural sites. Similarly, because large animals suffer less from cold and food scarcity than small ones, we expect (4) FID to change with climate variation more strongly for small than for large species, i.e., with a negative interaction between climatic variables and body size. Finally, we expect (5) different responses in FID to climate variation depending on the position in the food chain. Positive effects of increasing temperature and precipitation on food condition, and hence increasing FIDs, would particularly be expected at low trophic levels (i.e. herbivores), whereas negative effects promoting FID decrease would be expected especially by predators. Opposite effects of climate change on FIDs would ultimately destabilize predator–prey interactions by altering optimal local levels of antipredator behaviour and their potential consequences for population trends and food web structure. Overall, the differential effects of climate on antipredator behaviour would translate into different predictions of animal responses to climate change because of the changing strength of biotic interactions along latitudinal and land-use gradients.

## Results

Mean spring temperatures increased by 0.5 ºC during 2006–2019, whereas rainfall showed no significant linear temporal trend. Temporal trends did not differ significantly among study localities (Supplementary Material Table S1, Fig. S1). We gathered 32,145 FID records for 229 species of birds in 3,247 site x year combinations (Supplementary Material Table S2). FIDs were twice as long on average in rural as in urban habitats (20.0 m ± 0.7 m vs. 9.7 m ± 0.4 m; weighted means), habitat type having the largest effect size (Supplementary Material Table S3). FID increased with body mass and decreased northwards, with intermediate effect sizes (Table S3). FID for birds of prey was 1.5 times larger on average than for piscivores, and 2.2–2.4 times larger than for aerial feeders, other insectivores, herbivores and omnivores (Fig. S2), with an intermediate effect size.

**Figure 1:**
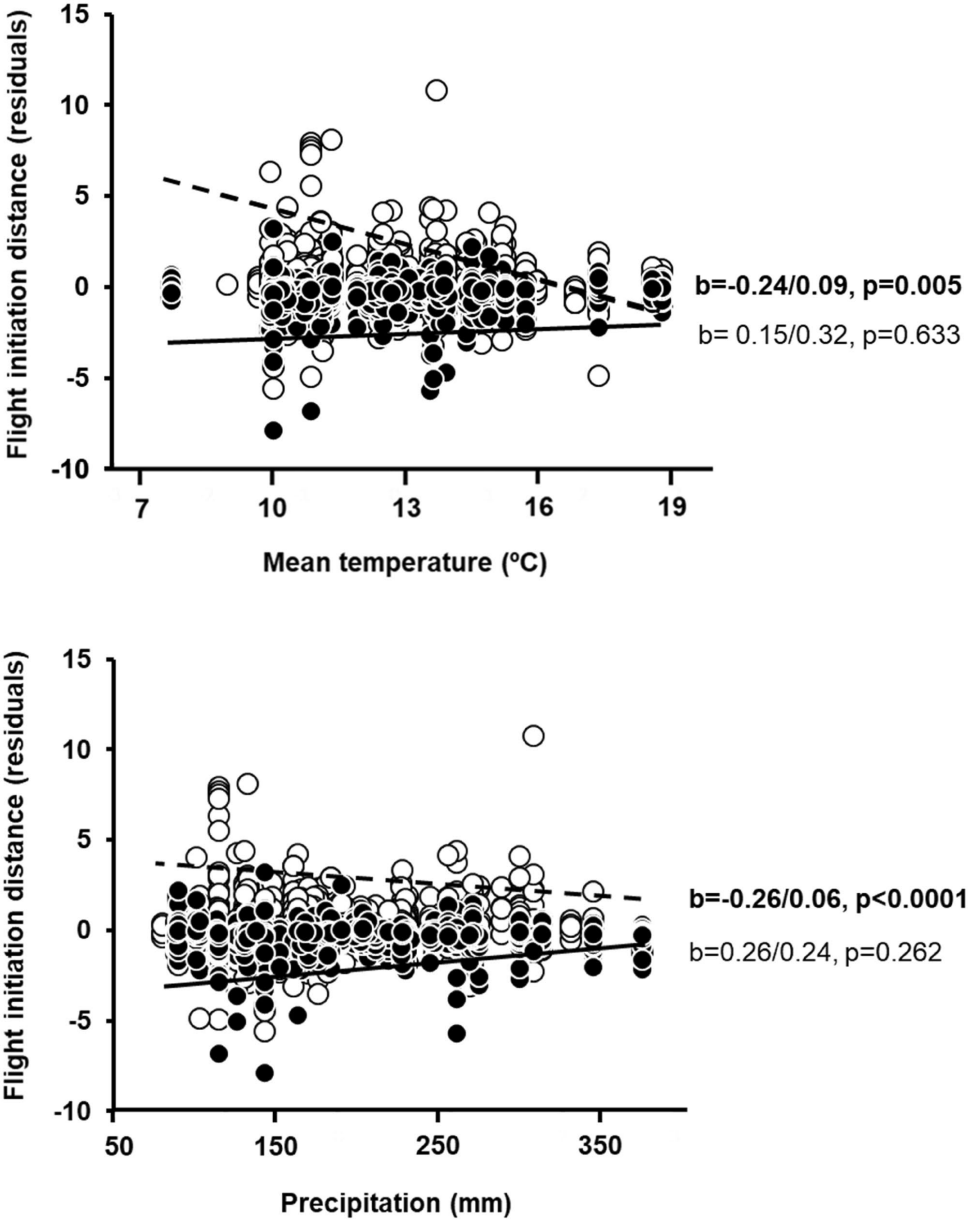
Relationships among mean spring temperature or precipitation during the local breeding season, and mean flight initiation distances (FID, corrected for the effects of species, site, year, latitude, body mass and diet) according to habitat (filled dots, continuous line: urban; open dots, dashed line: rural). Standardized regression coefficents (β/SE) and their p values are also given. Significant effects (p < 0.05) are in bold.

**Figure 2:**
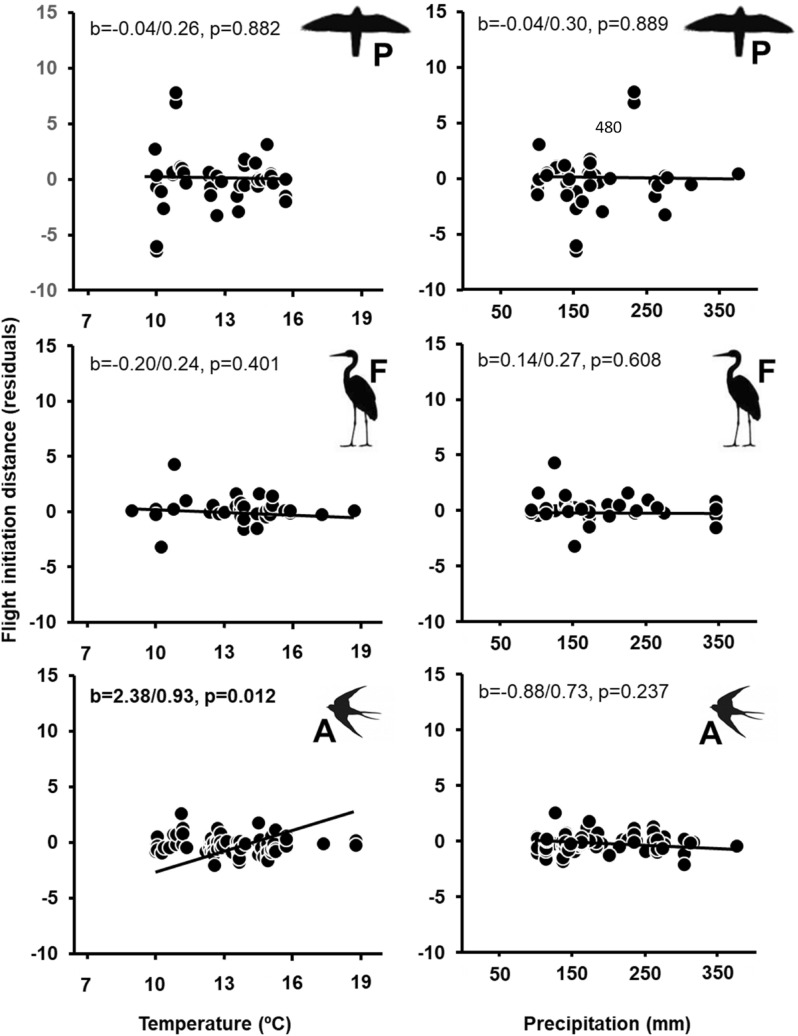

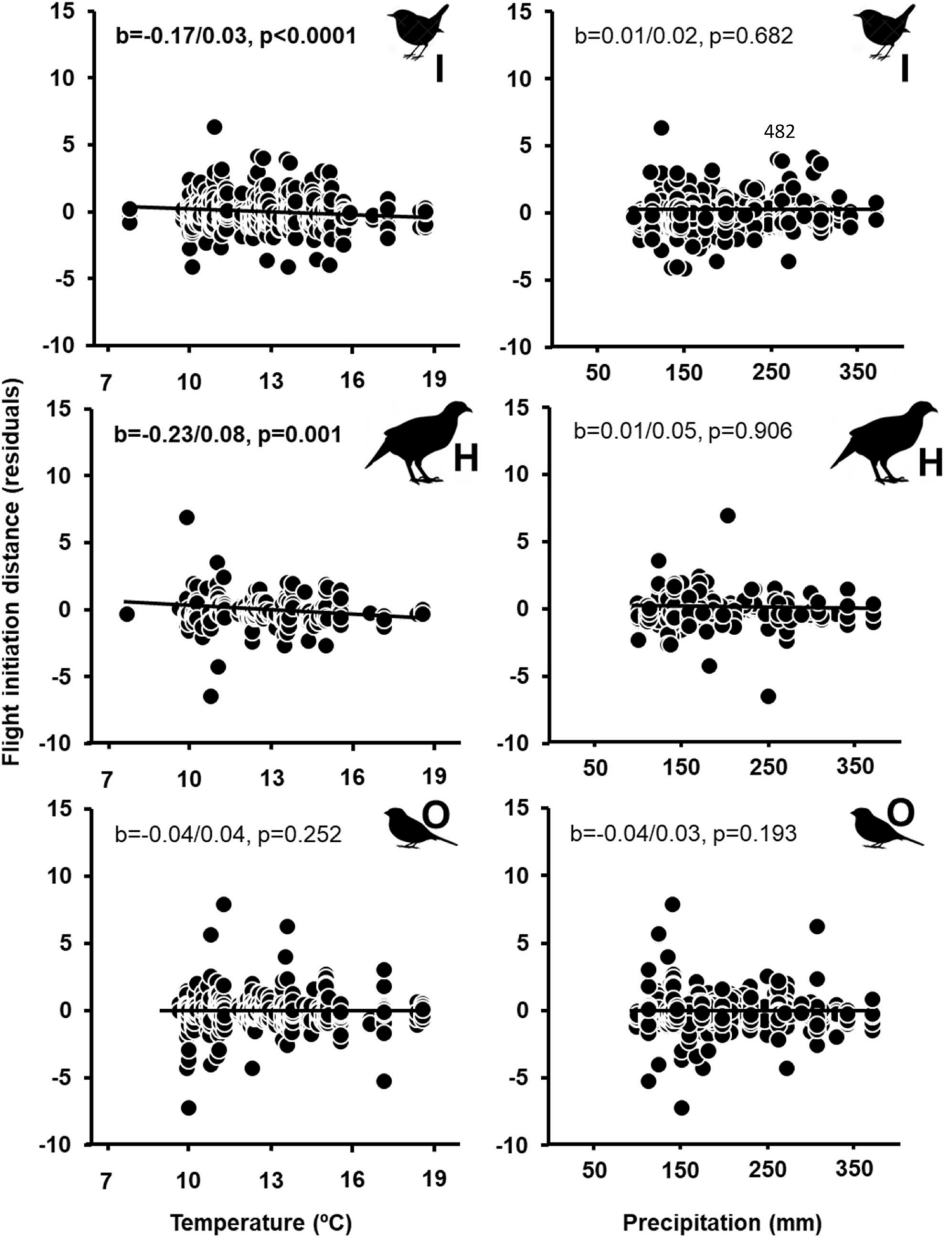
Relationships among mean spring temperature or precipitation during the local breeding season, and mean flight initiation distances (FID, corrected for the effects of species, site, year, latitude and body mass) in rural habitats according to diet (*P* predator, *F* piscivore, *I* insectivore, *A* aerial feeder, *H* herbivore, *O* omnivore; silhouettes of representative species of each group are included). Standardized regression coefficents (β/SE) and their p values are also given. Significant effects (p < 0.05) are in bold.

Higher spring temperature and lower precipitation were significantly associated with shorter FIDs, with small effect sizes (Supplementary Material Table S3). FIDs were shorter on average as temperature and rainfall increased. The correlation between climate and FID increased northwards (positive interaction with latitude) and it was weaker for larger species (negative interaction with body mass; both interactions had intermediate effect sizes). Climate effects on FID were significant in rural but not in urban habitats (Fig. 1). In rural habitats, FID-precipitation relationships were not significant for any diet group. Increasing temperature was related to longer FIDs in aerial feeders but with shorter FIDs in insectivores and herbivores. Predators, piscivores and omnivores showed non-significant negative trends (Fig. 2). These results were qualitatively similar when considering all data.

## Discussion

In order to survive and reproduce, all organisms must eat and avoid being eaten. Flight initiation distance (FID) measures how animals weigh foraging needs against predation risk^17^. Here we show that this behavioural response to changing environmental conditions was sensitive to climate variation and modulated by habitat, latitude, body mass and position in food webs. We propose that our findings on geographical patterns of FID variation can help predict how predator avoidance behaviour will respond to future global changes in climate and land use that will alter food availability, metabolic needs and predator abundance.

Globally our findings reinforce previous ones with FID shorter at higher latitudes and in urban compared with rural habitats in Europe^13^, and longer FIDs for larger species^14,28^. Longer FIDs as temperature and rainfall increased supported the model of lower foraging success under warmer and more humid climatic conditions, rather than the model based on climate effects on food abundance and needs^25,26^.

Effect sizes of these climate-behavior relationships were however small as compared to effects of latitude, habitat, body mass and diet. In fact, climate-behavior relationships were strongly modulated by these factors. Interactive effects of latitude were consistent with latitudinal change in the relative importance of biotic interactions^13,27^. Adaptive values of reduced FID would be higher when food conditions are more limited by climate and when low predation pressure allows for such reductions, as occurs northwards in Europe^13,15^. Urbanisation suppressed FID responses to climate variables. We hypothesize that more stable and favourable food and weather conditions within cities, as well as reduced predation risk, can account for this result^13,15,16,29^. Larger body mass mitigated climate associations with FID, as expected if larger birds suffer less from cold and food scarcity than small ones^30^. Body mass-predation risk relationships could also account for this mitigation effect, as larger birds would be less exposed to the small generalist predators usually found in and around cities^31,32^.

Finally, climate-driven FID change differed according to a species’ position in food webs. We found significant positive effects of mean temperature for aerial feeders, as expected from food availability hypotheses; negative effects expected from accessibility models for insectivores and herbivores; and not significant effects for predators, piscivores and omnivores. No significant effects of precipitation on FID were found. Differential climate change effects on FIDs at different trophic levels imply that expected effects of climate change on species may be modulated by changes at other trophic levels in more complex ways than expected from niche-based approaches^8,9^.

Behavioral responses to predation risk tend to have strong effects on prey populations besides pure numerical effects of predation^33^. Fear levels have in fact been found to be related to range and population trends^22,23^, although the strength and even direction of FID-trend relationships vary geographically according to general levels of human disturbance^22^. Highly variable fear responses to climate, and changing fearfulness-population trend relationships, imply a high degree of uncertainty when trying to understand how and why changes in biotic interactions may modify predictions from species-specific niche models. For instance, buffering effects of low latitude on fear responses may explain spatial patterns of expected resilience to climate change of urban bird communities in Europe, which have been shown to be potentially more resilient southwards despite climate change expectations^34^. Differential behavioural responses found here may provide the mechanistic basis for developing, and testing, refined models for the responses of bird communities to global change by modulating metabolism-based responses to abiotic and land use environmental factors.

## Methods

**Figure 3:**
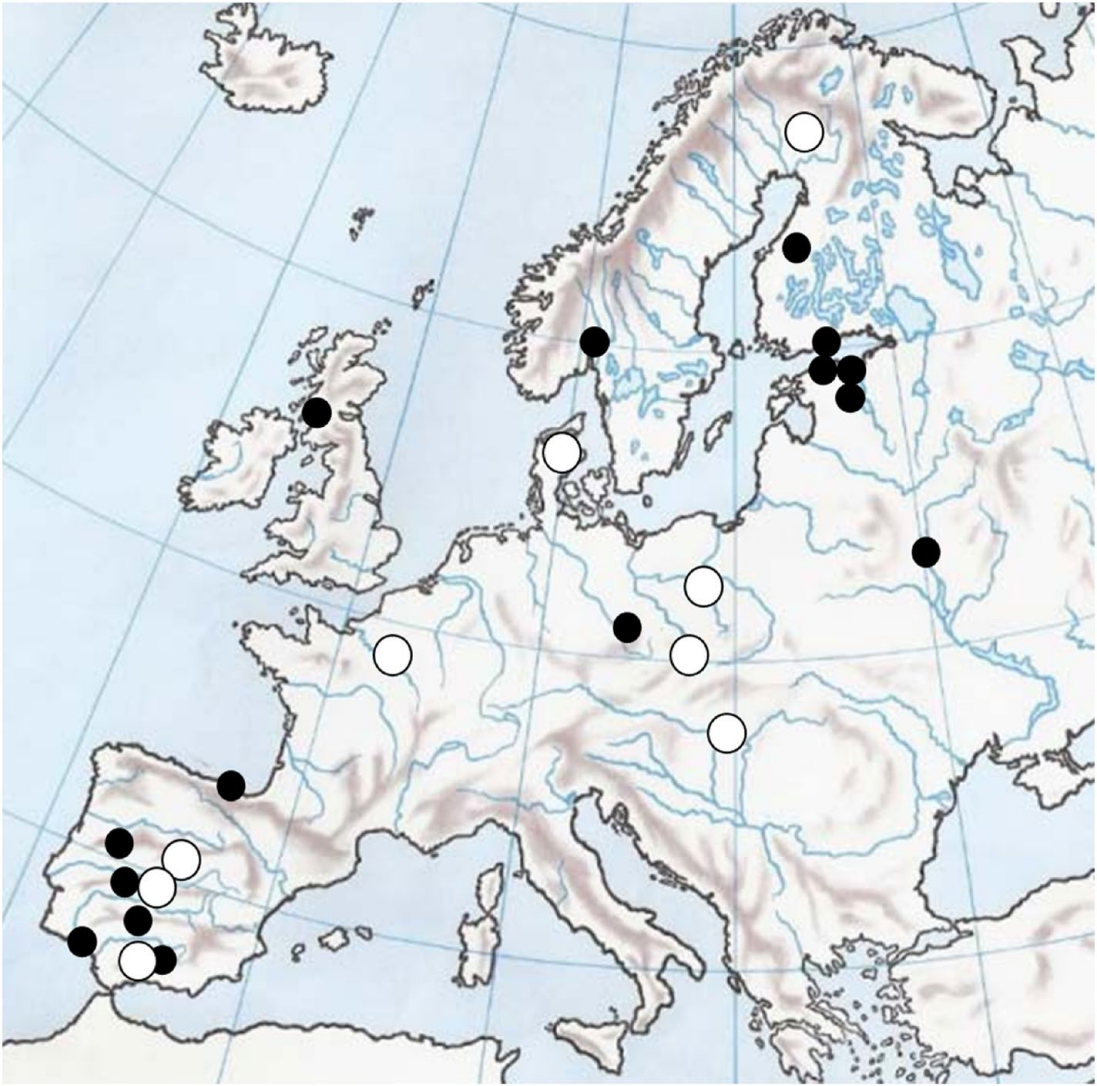
Location of the 24 study sites used for the study of flight initiation distance (FID) in Europe. Larger white spots indicate the nine study sites with more than 1000 FID records^13^. For latitudes and city names, see Supplementary Table S2. Map modified from https://www.pequeocio.com/wp-content/uploads/2018/04/mama-mudo-fisico-europa-768x551.jpg.

We collected data on flight initiation distances (FID, the distance at which an individual flees when approached by a human under standardized conditions, taken with a precision of 1 m^12^) between 2006 and 2019 in 24 European localities (Fig. 3). Most field work was done in paired urban and nearby rural sites to account for urbanization effects on fear responses^13,15^, attempting to obtain more than 300 FID records per site from as many species as possible. This meta-replicated approach across species, sites and habitats was established to ensure proper scientific generalization of results, even at the expense of weaker relationships associated with statistical noise (references in^29^). Trained observers approached individual birds previously detected by eye or with binoculars from at least 30 m away and the distance at which the bird flew away was recorded. These standardized procedures eliminate inter-observer effects as well as effects of starting distances, i.e., the distance at which the observer detects the bird and starts approaching it, on FID^13,15^. Data were gathered during the breeding season, avoiding individuals clearly engaged in nest building or offspring provisioning for ethical reasons, as well as recently fledged birds, less experienced than adults. Random sampling of birds mainly at their foraging sites made these observations rare. Species, sex and age when possible, were recorded. Only one individual per sex, age and species was recorded at specific sites within each location to avoid gathering repeated samples from the same individual. We calculated mean FID for each species in each locality and year (Supplementary Table S2), assuming that the effects of age and sex were negligible^15^. FID data were recorded by the following researchers in each region: Spain (MD, APM, JDIA), France, UK, Denmark, Norway and Ukranie (APM), Czech Republic (TG, FM), Hungary (GM), Poland (PT), Estonia (KT) and Finland (JJ, M-LK-J, APM).

Mean body mass (g) and main diet during the breeding season were taken from the most recent and comprehensive literature review for European birds^35^. Diets considered were herbivores (plants), insectivores (insects taken from surfaces), aerial feeders (insects taken in flight); piscivores (fish and other aquatic prey), predators (vertebrates), and omnivores (more than one of the categories above).

Climate data were obtained from the Essential Climate Variables database of the Copernicus project (https://cds.climate.copernicus.eu/cdsapp#!/dataset/ecv-for-climate-change?tab=overview). This database compiles smoothed climatic data at monthly intervals with a spatial resolution of 0.25° × 0.25°. Mean monthly temperature and overall rainfall were downloaded for the grid cells corresponding to each study site. Mean temperature and total precipitation for the breeding season of each study year were obtained by either averaging or summing up data from the three months covering the main breeding season for birds in each European city and their surroundings^29^. March–May was established for Western Europe (Spain, France and the UK), April-June for Central Europe (Czech Republic, Hungary, Poland and Ukranie), May–July for Southern Scandinavia (Denmark, Norway and Southernmost Finland) and Estonia, and June–August for Northern Finland.

Relationships among climate, geographic and bird trait variables were tested using Generalized Mixed Models (GLMMs) with mean FIDs as the dependent variable, weighted for sample sizes^36^. Logarithmic link functions and Gaussian distribution of errors were chosen on the basis of previous literature and inspection of the distributions of variables and residuals. Year was included as a fixed, continuous variable to control for the confounding effect of time, as FIDs are expected to decrease over time at least in urban settings because of the positive effect of reduced FID on avian fitness^23,24^. Species nested within localities were included as a random factor. We did not include formal phylogenetic control in the analyses because model convergence was precluded by strongly unbalanced designs, and because phylogenetic signals have proven to be weak or not significant in previous studies of FID^13,15^. We tested whether patterns of phylogenetic relatedness would have biased results by analysing the phylogenetic structure of model’ residuals by means of a mixed linear model with Order as the fixed factor and Species nested in Genus nested in Family nested in Order as the random factor^37^ (taxonomy followed birdsoftheworld.org/bow/home). No significant phylogenetic bias was found, as there were no effect of the fixed structure (F = 0.68, p = 0.832, df = 18, 518.98) and the random structure explained less than 0.1% variance (0.099%, SE = 0.023). We tested effects of temperature and precipitation on mean FID, together with main and interactive effects of latitude, habitat (rural or urban), body mass and diet. Preliminary analysis showed no interactive effects between temperature and precipitation. We used type III (orthogonal) sums of squares, and the Satterthwaite method to compute approximate degrees of freedom because of unbalanced data. Predictors were computed from the corresponding input variables by standardizing them to allow direct comparison of effect sizes and to make main effects biologically interpretable even when involved in interactions^38^. Statistical analyses were performed with SPSS 26.0. Effect sizes were computed as Pearson’s product–moment correlation coefficients from F values (t = $\sqrt{F}$)^39^ and judged small (r < 0.10), intermediate (r = 0.11–0.49) or large (r > 0.50) following previous recommendations^40^.

### Ethical statement

No permits are required for this kind of research in Europe. All methods used in the study were carried out in accordance with the approved guidelines. FID data collection is designed to cause only brief and minimal disturbance to birds, that does not differ from standard background disturbance caused by other visitors.

## Supplementary Information

Supplementary Table S2.

Supplementary Information.
