# Author Correction: Effects of climate variation on bird escape distances modulate community responses to global change

**DOI:** 10.1038/s41598-021-97174-x

**Published:** 2021-09-03

**Authors:** M. Díaz, T. Grim, G. Markó, F. Morelli, J. D. Ibáñez‑Alamo, J. Jokimäki, M.‑L. Kaisanlahti‑Jokimäki, K. Tätte, P. Tryjanowski, A. P. Møller

**Affiliations:** 1 Department of Biogeography and Global Change, Museo Nacional de Ciencias Naturales (BGC-MNCN-CSIC), c/Serrano 115bis, 28006 Madrid, Spain; 2 Department of Zoology and Laboratory of Ornithology, Palacky University, 77146 Olomouc, Czech Republic; 3 Behavioral Ecology Group, Department of Systematics, Zoology and Ecology, Eötvös Loránd University, Pázmány Péter sétány 1/c, 1117 Budapest, Hungary; 4 Department of Plant Pathology, Institute of Plant Protection, Hungarian University of Agriculture and Life Sciences, Ménesi út 44, 1118 Budapest, Hungary; 5 Faculty of Environmental Sciences, Community Ecology and Conservation, Czech University of Life Sciences Prague, Kamýcká 129, 165 00 Prague 6, Czech Republic; 6 Department of Zoology, Faculty of Sciences, University of Granada, 18071 Granada, Spain; 7 Nature Inventory and EIA‑Services, Arctic Centre, University of Lapland, P. O. Box 122, 96101 Rovaniemi, Finland; 8 Department of Zoology, Institute of Ecology and Earth Sciences, University of Tartu, 19 51014 Tartu, Estonia; 9 Institute of Zoology, Poznań University of Life Sciences, Wojska Polskiego 71C, 60625 Poznań, Poland; 10 Ecologie Systématique et Evolution, Université Paris-Saclay, CNRS, AgroParisTech, 91405 Orsay, France

Correction to: *Scientific Reports* https://doi.org/10.1038/s41598-021-92273-1, published online 18 June 2021

The original version of this Article contained an error in the upper panel of Figure 1, where the negative relationships among mean spring temperature or precipitation during the local breeding season were displayed as positive.

The original Figure 1 and accompanying legend appear below.

**Figure 1:**
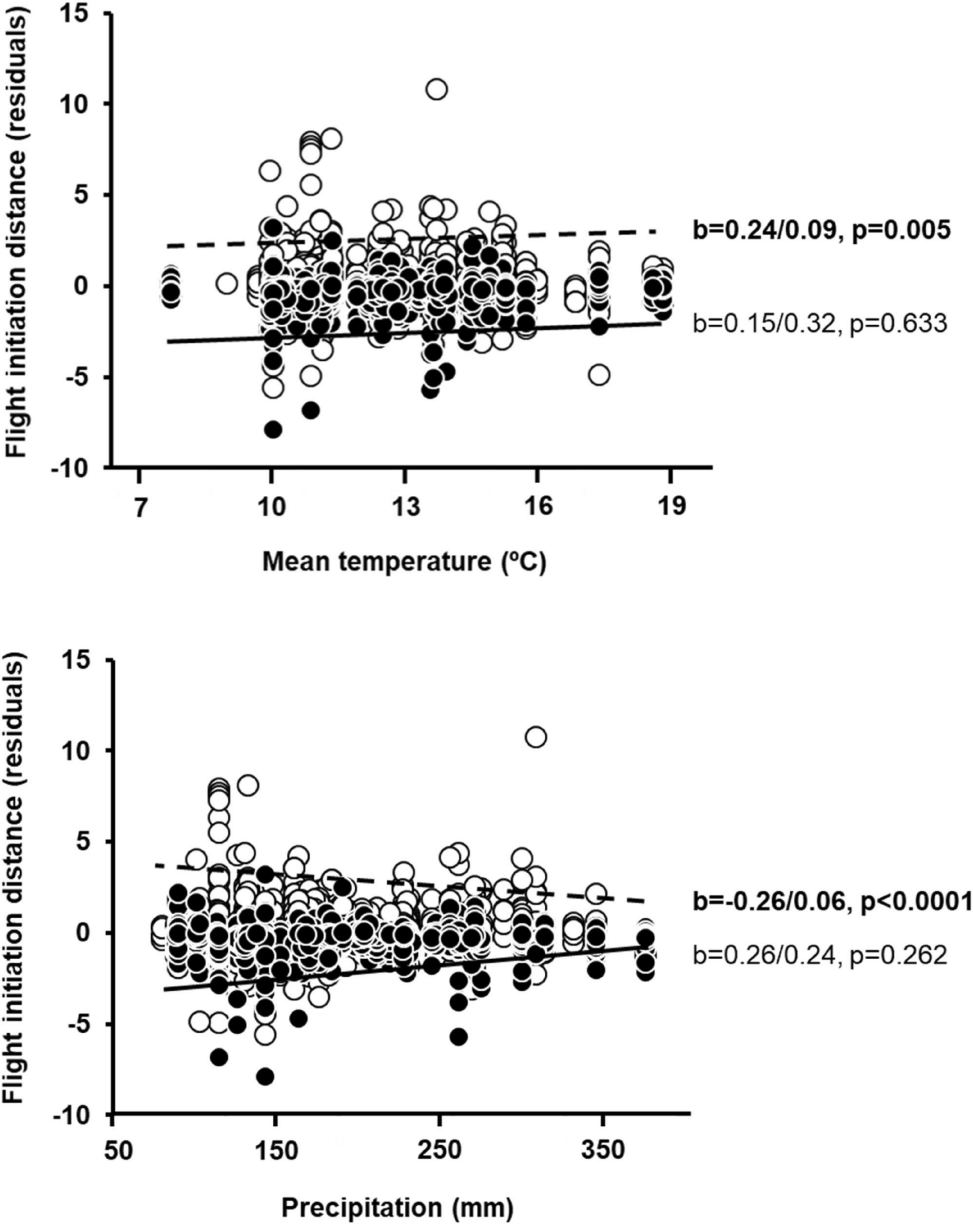
Relationships among mean spring temperature or precipitation during the local breeding season, and mean flight initiation distances (FID, corrected for the effects of species, site, year, latitude, body mass and diet) according to habitat (filled dots, continuous line: urban; open dots, dashed line: rural). Standardized regression coefficents (β/SE) and their p values are also given. Significant effects (*p* < 0.05) are in bold.

The original Article has been corrected.

