# Supplementary material for: Effects of climate variation on bird escape distances modulate community responses to global change
Source: Sci Rep. 2021 Jun 18;11:12826. doi: 10.1038/s41598-021-92273-1 (PMC8213824; doi:10.1038/s41598-021-92273-1)
Supplement: Supplementary file 2 — Supplementary Information. [file 41598_2021_92273_MOESM2_ESM.docx]

**Effects of climate variation on bird escape distances modulate community responses to global change**

**Díaz, M.^a*^, Grim, T. ^b^, Markó, G.^c,d^, Morelli, F.^e^, Ibáñez-Alamo, J.D.^f^, Jokimäki, J.^g^, Kaisanlahti-Jokimäki, M.-L.^g^, Tätte, K.^h^, Tryjanowski, P.^i^, Møller, A.P.^j^**

^a^ Department of Biogeography and Global Change, Museo Nacional de Ciencias Naturales (BGC-MNCN-CSIC), c/Serrano 115bis, E-28006 Madrid, Spain, [Mario.Diaz@ccma.csic.es](mailto:Mario.Diaz@ccma.csic.es)

^b^ Palacky University, Department of Zoology and Laboratory of Ornithology, CZ-77146 Olomouc, Czech Republic

^c^ Eötvös Loránd University, Behavioral Ecology Group, Department of Systematics, Zoology and Ecology, Pázmány Péter sétány 1/c, H-1117 Budapest, Hungary

^d^ Department of Plant Pathology, Institute of Plant Protection, Hungarian University of Agriculture and Life Sciences , Ménesi út 44, H-1118 Budapest, Hungary

^e^ Czech University of Life Sciences Prague, Faculty of Environmental Sciences, Community Ecology & Conservation, Kamýcká 129, CZ-165 00 Prague 6, Czech Republic

^f^ Department of Zoology, Faculty of Sciences, University of Granada, E-18071 Granada, Spain

^g^ Nature Inventory and EIA-services, Arctic Centre, University of Lapland, P. O. Box 122, FI-96101 Rovaniemi, Finland

^h^ University of Tartu, Institute of Ecology & Earth Sciences, Department of Zoology, EE-19 51014 Tartu, Estonia

^i^ Institute of Zoology, Poznań University of Life Sciences, Wojska Polskiego 71C, PL-60-625 Poznań, Poland,

^j^ Ecologie Systématique et Evolution, Université Paris-Saclay, CNRS, AgroParisTech, 91405, Orsay, France.

**Additional information**

**Supplementary Material Table S1** Results of ANCOVAs testing for spatial and temporal trends in climate variables. Significant effects (p<0.05) are in bold. Two localities (Santander and Andújar) were excluded from analyses because of missing data. March-May data for all years and study localities were used for consistency in the analysis of climate trends.

|  |  | Mean temperature | |  |  | Overall precipitation | |
| --- | --- | --- | --- | --- | --- | --- | --- |
| Effect | df | F | p |  |  | F | p |
| Year | 21 | 6.15 | **0.014** |  |  | 0.15 | 0.703 |
| Locality | 1 | 215.46 | **<0.001** |  |  | 5.20 | **<0.001** |
| Year x Locality | 21 | 0.20 | 1.000 |  |  | 0.32 | 0.998 |
| Error | 264 |  |  |  |  |  |  |
|  |  |  |  |  |  |  |  |
| Model | 43 | 105.46 | **<0.001** |  |  | 2.70 | **<0.001** |
| Adjusted R^2^ |  | 93.60 |  |  |  | 19.22 |  |

**Supplementary Material Table S2** Summary statistics for flight initiation distances (FID) collected for 229 bird species between 2006 and 2019 at 24 European localities. Geographical coordinates, habitat (urban or rural) and climate data during the local breeding season are given for each site. Body mass and main diet during the breeding season (P: predator; F: piscivore; I: insectivore; A: aerial feeder; H: herbivore; O: omnivore) for each species are also given. See text for details.

**Supplementary Material Table S3** Results of GLMMs testing for climate effects on flight initiation distances of European birds. R: rural; U: urban; P: predator; F: piscivore; I: insectivore; A: aerial feeder; H: herbivore and O: omnivore. Significant effects (p<0.05) are in bold. Effect sizes are Pearson’s r values computed from F and df values^37^. Variance explained by the random term (species nested within localities) was 28.2% (p<0.0001).

| Effect | F | df | p | b(SE) | effect size |
| --- | --- | --- | --- | --- | --- |
| Year | 0.02 | 1, 3149 | 0.889 | 0.00(0.00) | 0.003 |
| Habitat | 648.13 | 1, 2873 | **<0.001** | R>U | 0.429 |
| Latitude | 31.86 | 1, 1170 | **<0.001** | -0.13(0.02) | 0.163 |
| Body mass | 129.9 | 1, 718 | **<0.001** | 0.19(0.02) | 0.392 |
| Diet | 11.07 | 5, 836 | **<0.001** | P>F>A=I=H=O | 0.114 |
|  |  |  |  |  |  |
| Temperature | 13.13 | 1, 2415 | **<0.001** | -0.05(0.07) | 0.074 |
| Precipitation | 11.98 | 1, 3198 | **<0.001** | -0.23(0.05) | 0.061 |
|  |  |  |  |  |  |
| Habitat x Temperature | 4.86 | 1, 2966 | **0.024** | no significant climate effects in urban sites | 0.040 |
| Habitat x Precipitation | 12.23 | 1, 2844 | **<0.001** |  | 0.065 |
|  |  |  |  |  |  |
| Latitude x Temperature | 99.7 | 1, 2727 | **<0.001** | 0.22(0.02) | 0.188 |
| Latitude x Precipitation | 155.32 | 1, 3162 | **<0.001** | 0.19(0.02) | 0.216 |
|  |  |  |  |  |  |
| Body mass x Temperature | 29.92 | 1, 2093 | **<0.001** | -0.06(0.01) | 0.119 |
| Body mass x Precipitation | 85.12 | 1, 3087 | **<0.001** | -0.07(0.01) | 0.164 |
|  |  |  |  |  |  |
| Diet x Temperature | 3.21 | 5, 2107 | **0.009** | Different slopes according to diet | 0.038 |
| Diet x Precipitation | 3.39 | 5, 2998 | **0.002** |  | 0.034 |
|  |  |  |  |  |  |
| Model | 48.23 | 27, 1896 | **<0.001** | AICc=9861.7 |  |

**Supplementary Material Fig. S1** Trends in March-May mean temperature (above) and overall precipitation (below) for 22 out of the 24 study sites during 2006-2019. See also Table S1.

**Supplementary Material Fig. S2** Mean (±SE –box- and 95%CI -whiskers-) flight initiation distances, weighted for the effects of species, site, year, latitude, body mass and climate effects, according to bird’s main diet^34^. Sample sizes (No. populations/No. species) are indicated under silhouettes of representative species of each group.

**
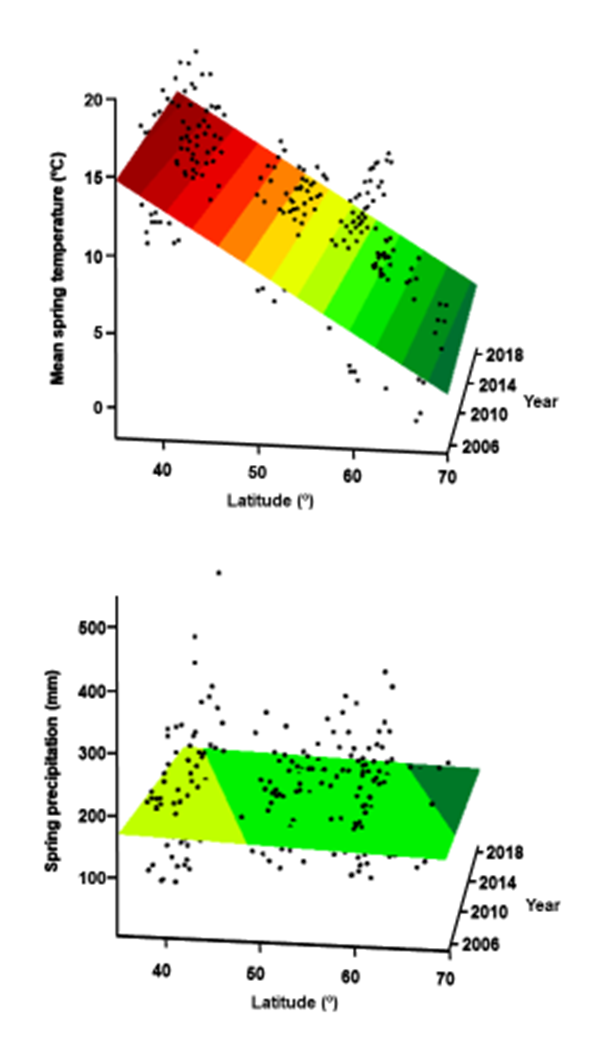
Fig. S1**

**
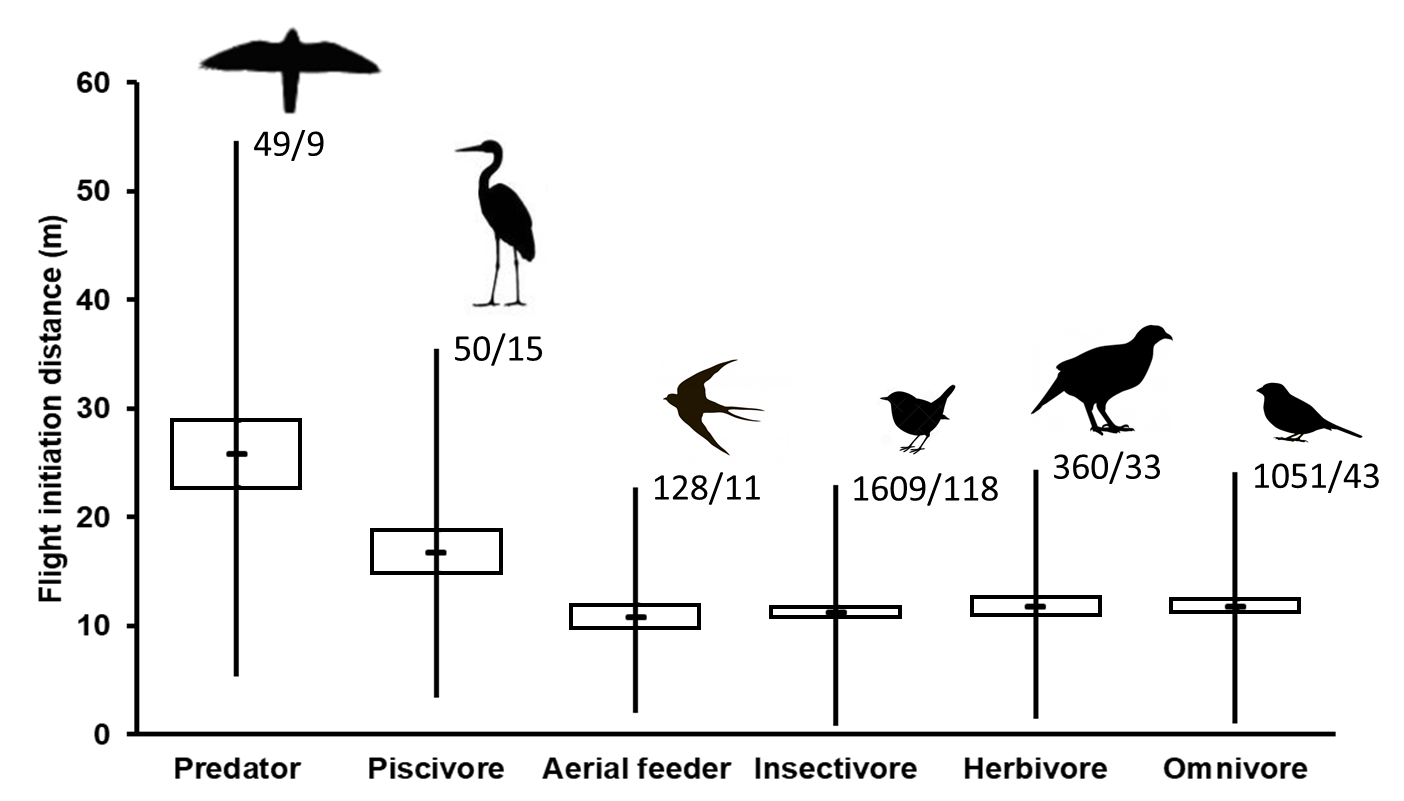
Fig. S2**
